# A Self-Inserted Foreign Body in the Urinary Bladder and Urethra

**DOI:** 10.7759/cureus.16322

**Published:** 2021-07-11

**Authors:** Hakan Tuncer, Hatice Karacam, Betul Cam

**Affiliations:** 1 Emergency Medicine, Bağcılar Training and Research Hospital, Istanbul, TUR

**Keywords:** urinary bladder, urethra, radiology, physical examination, foreign body

## Abstract

Foreign objects in the lower genitourinary system are a rare urological emergency often associated with self-eroticism, drug intoxication, or psychiatric illness. In addition to clinical examination, multiple imaging modalities such as X-ray, ultrasound, computed tomography, and magnetic resonance imaging have been used for the diagnosis of foreign bodies. Surgical exploration and endoscopic extraction are the main approaches to the treatment. Here, we present the case of a 37-year-old male who presented to the emergency department with penile and urethral pain caused by an electrical wire inserted into the urethra. The electrical wire was protruding 15 cm from the urethral meatus. A 50 cm long cable was extracted from the urethra and urinary bladder under regional anesthesia. This case is remarkable for the length of the foreign body and the depth to which it was inserted reaching into the urinary bladder. Emergencies related to sexuality or unconventional sexual preferences can lead to avoidance or delay of medical treatment, which, in turn, can result in a higher risk of complications. The examining doctor should be sensitive to secretive and insecure behavior and should be considerate of the patient’s privacy to facilitate a thorough physical examination.

## Introduction

The most common complaints associated with foreign objects in the genitourinary organs include pelvic pain, hematuria, dysuria, urinary frequency, strangury, urinary retention, and secondary stones [1-7]. A wide variety of objects have been reported to be self-inserted into the lower genital organs such as pins, electrical wires, screws, olive seeds, or ballpoint pens [2-6]. In 63% of such cases, the patients are males of different ages [8]. Clinical history, physical examination, and imaging tests help physicians diagnose foreign objects in the human body [9,10]. Some of the behavior resulting in the self-insertion of such objects is caused by mental health illness, senility, drug intoxication, and autoerotic stimulation [7]. In non-psychotic mental disorders where sexual gratification is achieved by unusual and potentially harmful actions, habitualization is common [11,12]. Here, we report the case of a 37-year-old male patient who presented to our Emergency Department with transurethral insertion of a 50 cm long electrical wire into the urinary bladder.

## Case presentation

**Figure 1:**
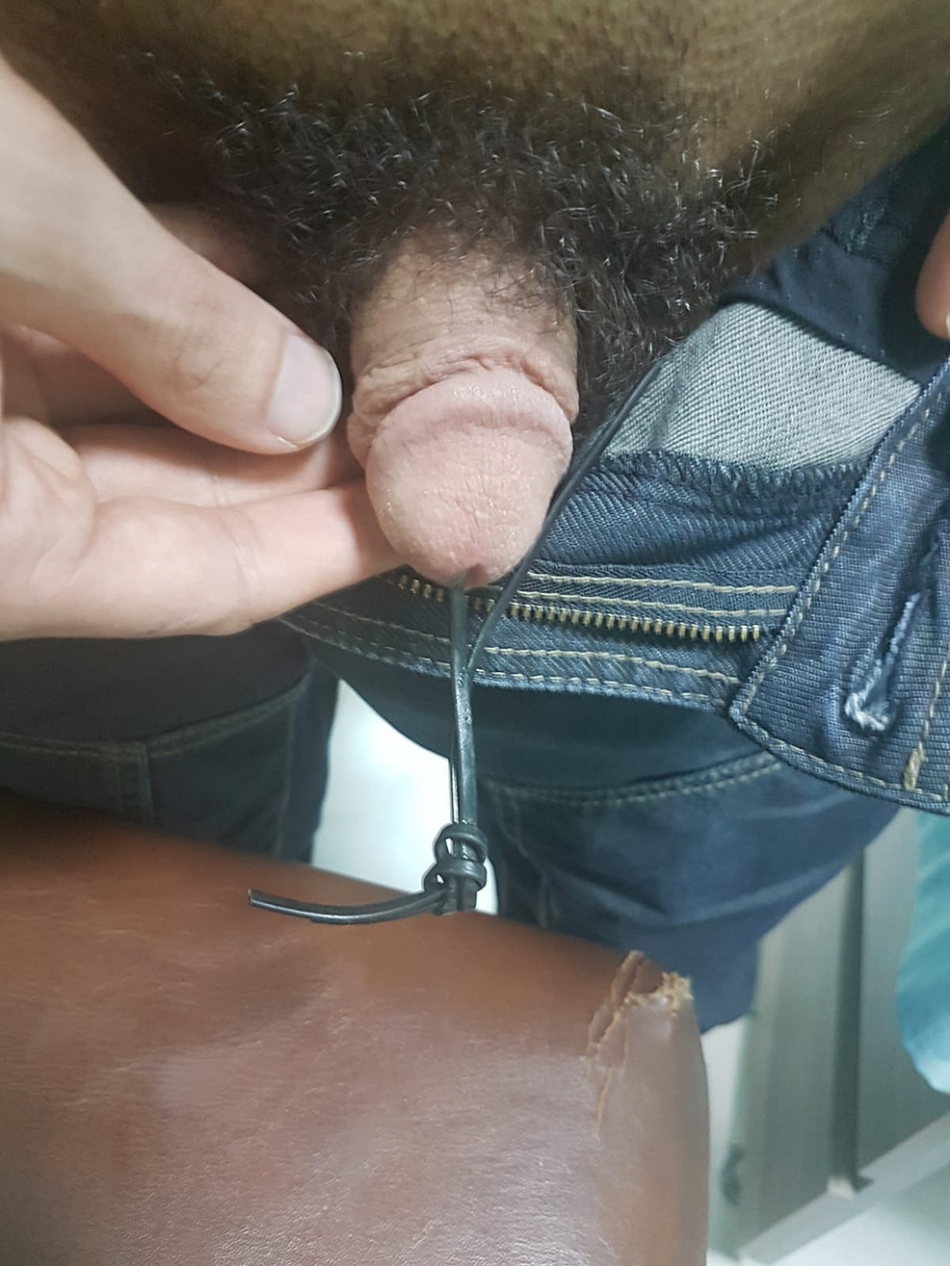
A foreign body in the urethra.

**Figure 2:**
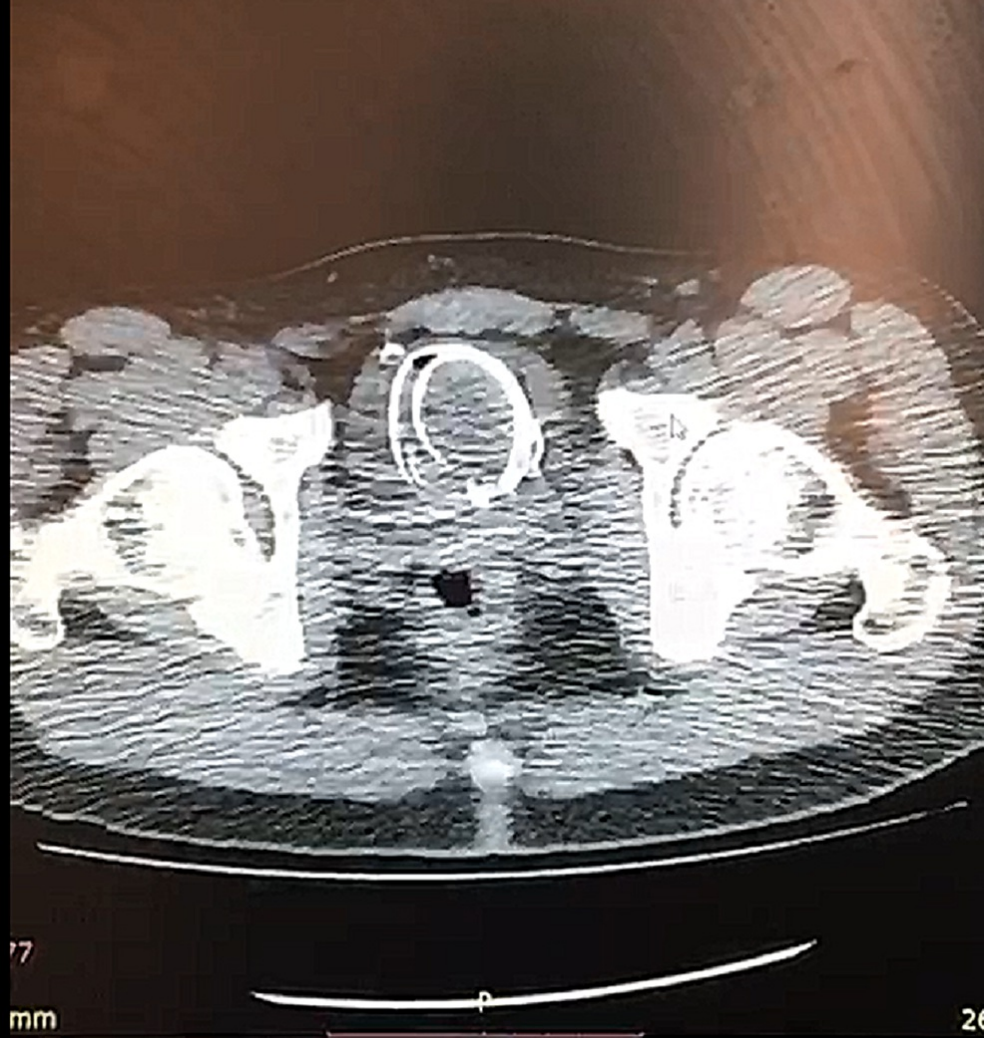
The cable demonstrates a circular shape in the urinary bladder.

A 37-year-old male patient presented to the Emergency Department complaining of severe penile pain. Physical examination revealed an electrical wire protruding 15 cm from the urethral meatus (Figures 1, 2).

**Figure 3:**
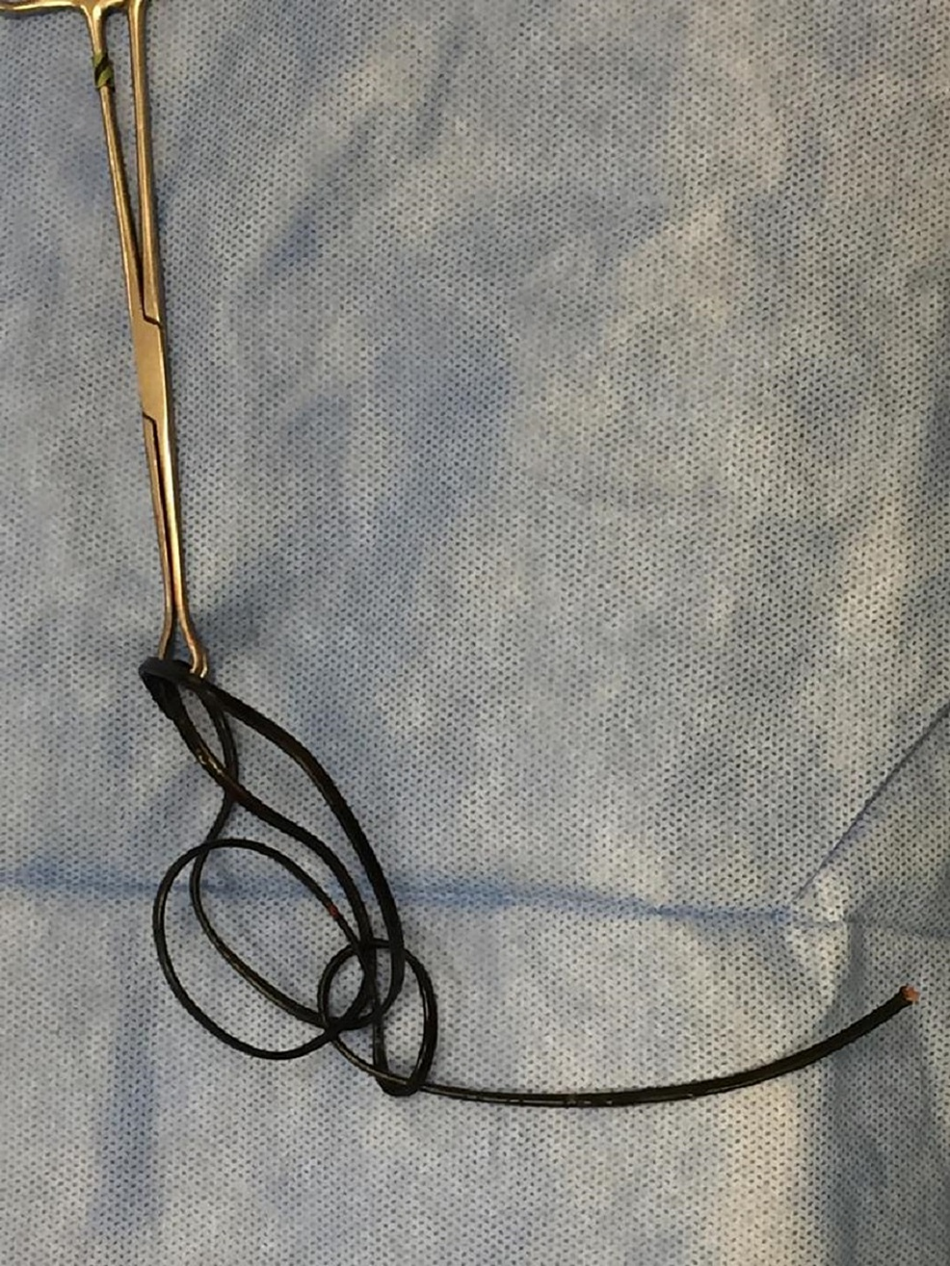
The extracted electrical wire.

The patient had no significant medical history. He disclosed the reason for the self-insertion of the wire as sexual arousal. A non-enhanced computed tomography scan (CT) of the pelvic cavity revealed a circular wire inside the urinary bladder. A urethral extraction was immediately scheduled by the urologist and the wire was extracted under local anesthesia (Figure 3).

The wire had a total length of about 65 cm, 50 cm of which had been lodged inside the body. A follow-up plan was scheduled with the urology and psychiatric departments.

## Discussion

Several different foreign objects have been previously reported to be self-inserted into the lower genitourinary tract, such as cables, screws, nuts, metallic tongue cleaner, and pens [1-7]. Forcing such objects into bodily orifices can be harmful. X-ray, ultrasound, CT, and magnetic resonance imaging are the imaging tests of choice to determine the presence of such objects. X-rays and ultrasounds are cheaper and more accessible in many clinics [10]. Endoscopic and surgical interventions are used to remove foreign bodies [13-16]. An endoscopic procedure is a fast and recommended method for removing foreign bodies under local anesthesia. Open surgery may be required for sharp and irregularly shaped objects. In our case, a looped circular wire was detected in the urinary bladder on a CT scan and was successfully removed under local anesthesia.

## Conclusions

Self-insertion of foreign bodies into the urinary tract is rather rare in emergency medicine practice. Imaging is necessary to determine the urethral location, shape, and size of the foreign objects. Urethral stenosis could develop following surgical interventions due to tissue damage. Tetanus prophylaxis and antibiotic treatment are required after the removal of the foreign body. Further, psychiatric counseling should be offered for habitual cases.
